# Supplementary material for: Common activation mechanism of class A GPCRs
Source: eLife. 2019 Dec 19;8:e50279. doi: 10.7554/eLife.50279 (PMC6954041; doi:10.7554/eLife.50279)
Supplement: Figure 2—source data 2. [file elife-50279-fig2-data2.pdf]

Figure 2—Source data 2. Thirty-four residue pairs shown conserved rearrangements of residue contacts upon activation.

This PDF file includes:

Auxiliary Supplementary Figs. S1 to S8

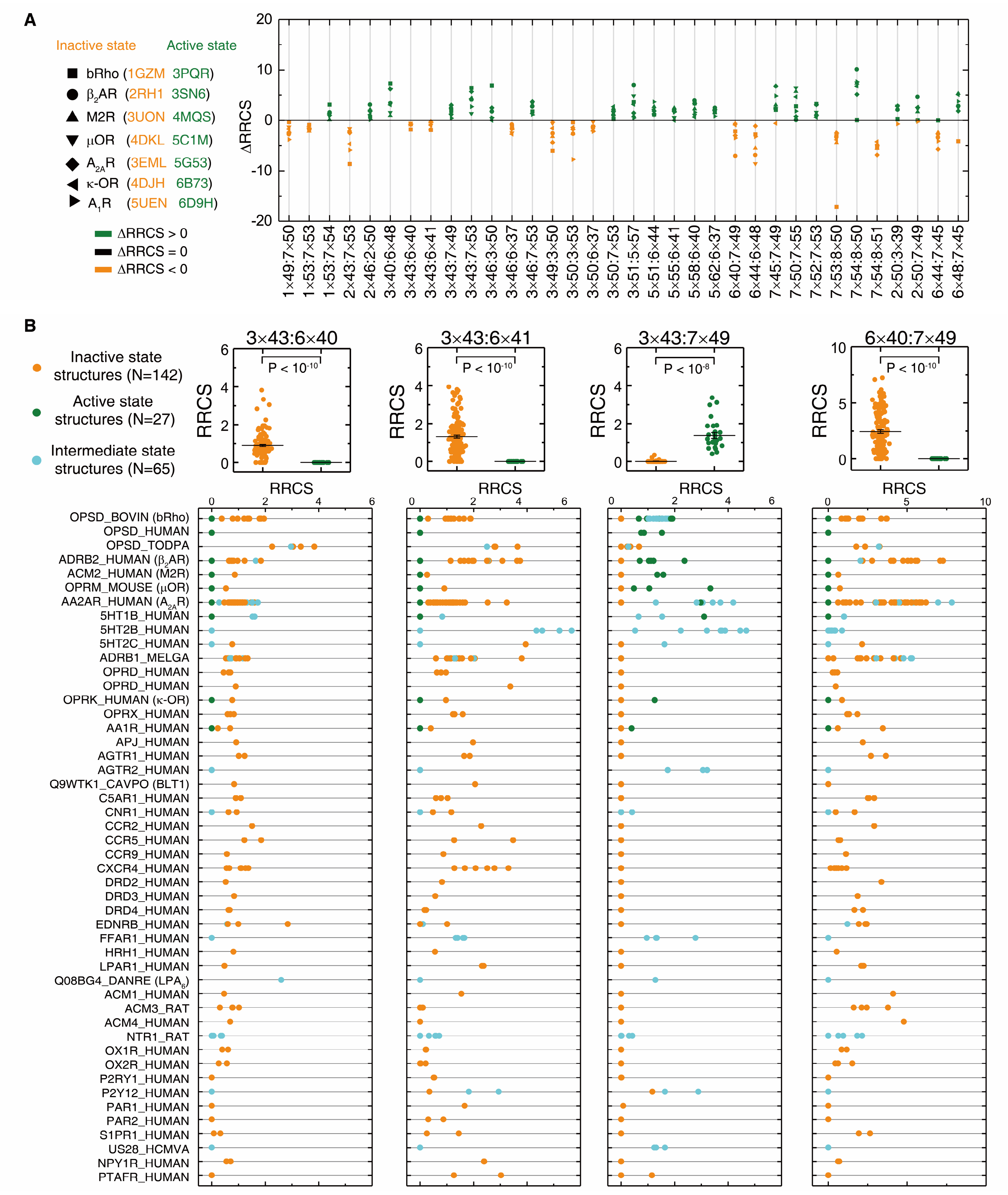
A
Inactive state
Active state
bRho (1GZM 3PQR)
β2AR (2RH1 3SN6)
M2R (3UON 4MQS)
μOR (4DKL 5C1M)
A2AR (3EML 5G53)
κ-OR (4DJH 6B73)
A1R (5UEN 6D9H)
ΔRRCS > 0
ΔRRCS = 0
ΔRRCS < 0
ΔRRCS
20
10
0
-10
-20
1×49:7×50
1×53:7×53
1×53:7×54
2×43:7×53
2×46:2×50
3×40:6×48
3×43:6×40
3×43:6×41
3×43:7×49
3×43:7×53
3×46:3×50
3×46:6×37
3×46:7×53
3×49:3×50
3×50:3×53
3×50:6×37
3×50:7×53
3×51:5×57
5×51:6×44
5×55:6×41
5×58:6×40
5×62:6×37
6×40:7×49
6×44:6×48
7×45:7×49
7×50:7×55
7×52:7×53
7×53:8×50
7×54:8×50
7×54:8×51
2×50:3×39
2×50:7×49
6×44:7×45
6×48:7×45
B
Inactive state structures (N=142)
Active state structures (N=27)
Intermediate state structures (N=65)
3×43:6×40
3×43:6×41
3×43:7×49
6×40:7×49
P < 10-10
P < 10-10
P < 10-8
P < 10-10
RRCS
OPSD_BOVIN (bRho)
OPSD_HUMAN
OPSD_TODPA
ADRB2_HUMAN (β2AR)
ACM2_HUMAN (M2R)
OPRM_MOUSE (μOR)
AA2AR_HUMAN (A2AR)
5HT1B_HUMAN
5HT2B_HUMAN
5HT2C_HUMAN
ADRB1_MELGA
OPRD_HUMAN
OPRD_HUMAN
OPRK_HUMAN (κ-OR)
OPRX_HUMAN
AA1R_HUMAN
APJ_HUMAN
AGTR1_HUMAN
AGTR2_HUMAN
Q9WTK1_CAVPO (BLT1)
C5AR1_HUMAN
CNR1_HUMAN
CCR2_HUMAN
CCR5_HUMAN
CCR9_HUMAN
CXCR4_HUMAN
DRD2_HUMAN
DRD3_HUMAN
DRD4_HUMAN
EDNRB_HUMAN
FFAR1_HUMAN
HRH1_HUMAN
LPAR1_HUMAN
Q08BG4_DANRE (LPA6)
ACM1_HUMAN
ACM3_RAT
ACM4_HUMAN
NTR1_RAT
OX1R_HUMAN
OX2R_HUMAN
P2RY1_HUMAN
P2Y12_HUMAN
PAR1_HUMAN
PAR2_HUMAN
S1PR1_HUMAN
US28_HCMVA
NPY1R_HUMAN
PTAFR_HUMAN

Fig. S1. Residue pairs with conserved rearrangements of residue contacts upon activation. (**A**) For 7 receptors with both inactive- and active-state structures available (Rho, β_2AR, M2R, μOR, A_{2A}R, κ-OR and recently determined A_1R), 34 intra-receptor residue pairs showed almost identical tendency of ΔRRCS upon activation, *i.e.*, their ΔRRCS are almost entirely positive or negative. Considering there is no sodium pocket for rhodopsin, four related residue pairs around sodium pocket (2×46:2×50, 2×50:3×39, 2×50:7×49 and 6×44:7×45) were neglected for bRho. (**B**) Four residue pairs (see Supplementary Figs. 2-8 for the rest 30 residue pairs) have significant different RRCS values between inactive- and active- states, *i.e.*, they evolve similar conformational changes upon receptor activation at the RRCS-level (two-sample *t*-test, error bars represent the standard error of the mean). The comparison of RRCS between inactive- and active-state were plotted in receptor-specific manner for 45 class A GPCRs with determined structures, where inactive, active and intermediate states are coloured in orange, cyan and green, respectively.

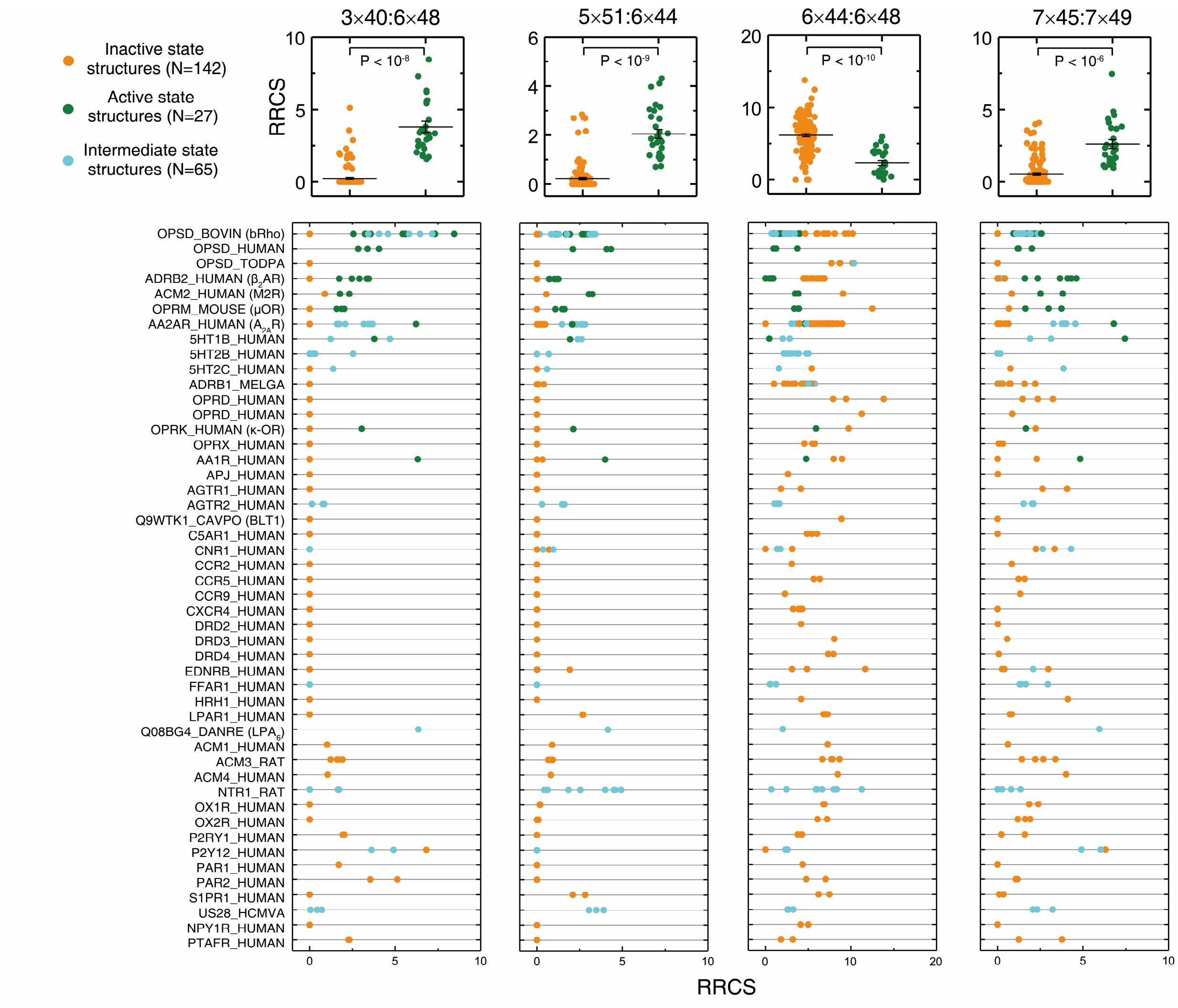

Fig. S2. Conserved signal initiation step involving changes in residue contacts at the bottom of the ligand-binding pocket. These residue pairs have significant different RRCS between inactive- and active- states, i.e., they evolve similar rearrangements of residue contacts upon activation (two-sample *t*-test, error bars represent the standard error of the mean). The comparison of RRCS between inactive- and active- state were plotted in receptor-specific manner for 45 class A GPCRs with determined structures, where inactive, active and intermediate states are coloured in orange, cyan and green, respectively.

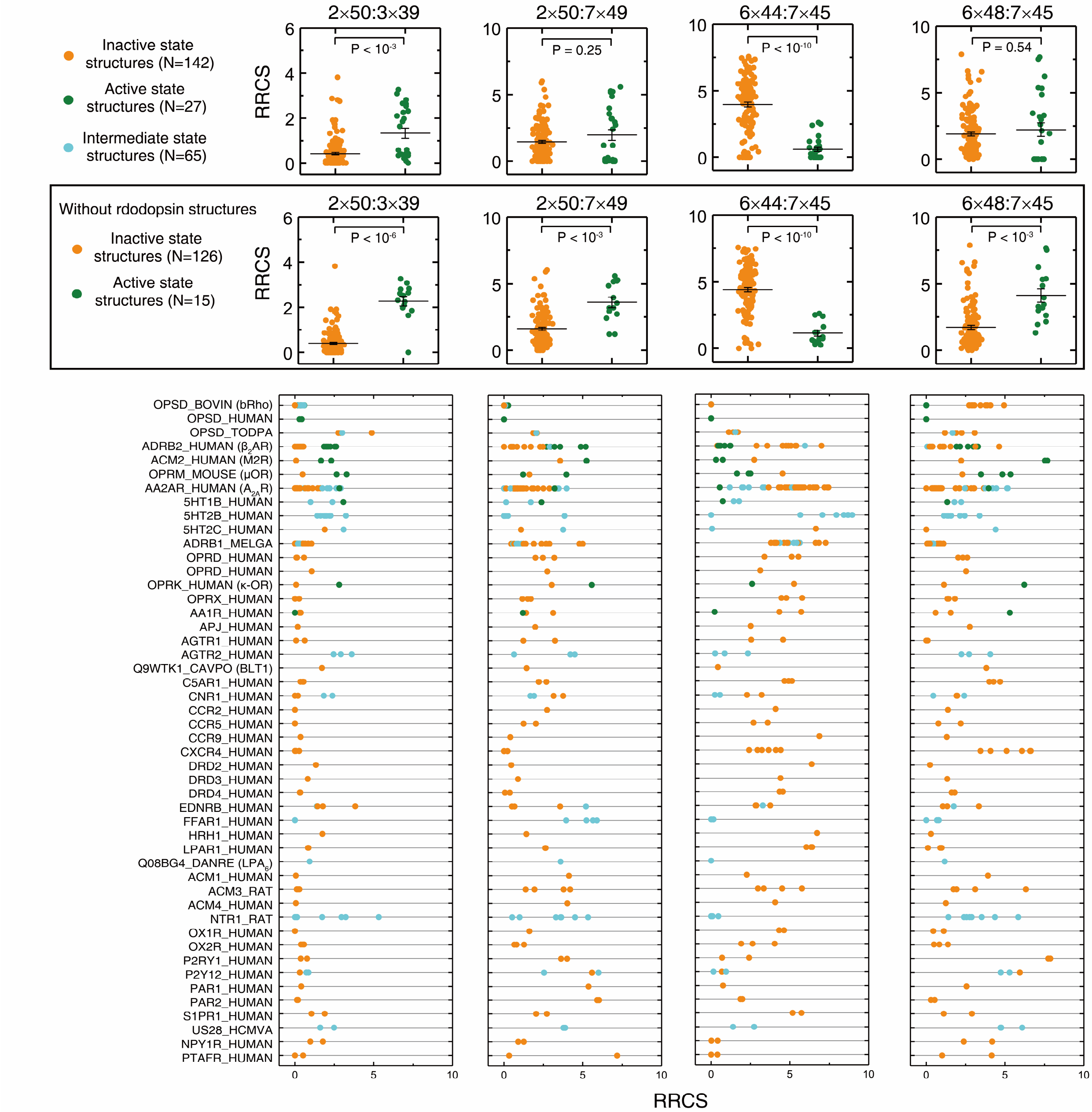

Fig. S3. Residue pairs around sodium pocket share conserved rearrangements of residue contacts upon activation. Considering there is no sodium pocket (residues at 2×50, 3×39, 7×45 and 7×49) for rhodopsin, four related residue pairs (2×46:2×50, 2×50:3×39, 2×50:7×49 and 6×44:7×45) were omitted for rhodopsin. These residue pairs have significant different RRCS between inactive- and active- state, i.e., they evolve rearrangements of residue contacts upon activation (two-sample *t*-test, error bars represent the standard error of the mean).

The comparison of RRCS between inactive- and active- state were plotted in receptor-specific manner for 45 class A GPCRs with determined structures, where inactive, active and intermediate states are coloured in orange, cyan and green, respectively.

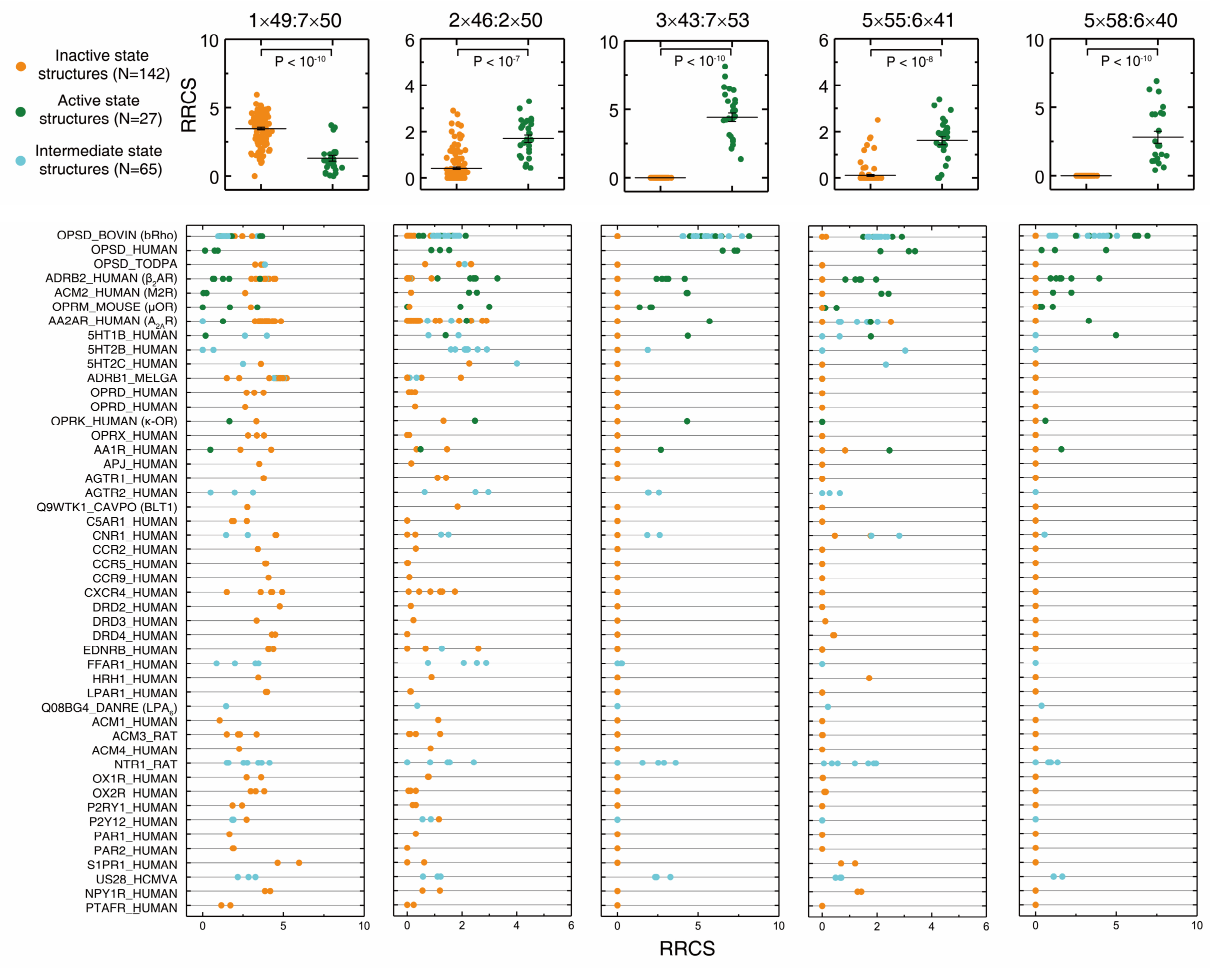

Fig. S4. Residue pairs around hydrophobic lock (3 × 43, 6× 40 and 6× 41) in layer 2 share conserved rearrangements of residue contacts upon activation. These residue pairs have significant different RRCS between inactive- and active- states, i.e., they evolve rearrangements of residue contacts upon activation (two-sample *t*-test, error bars represent the standard error of the mean). The comparison of RRCS between inactive- and active- state were plotted in receptor-specific manner for 45 class A GPCRs with determined structures, where inactive, active and intermediate states are coloured in orange, cyan and green, respectively.

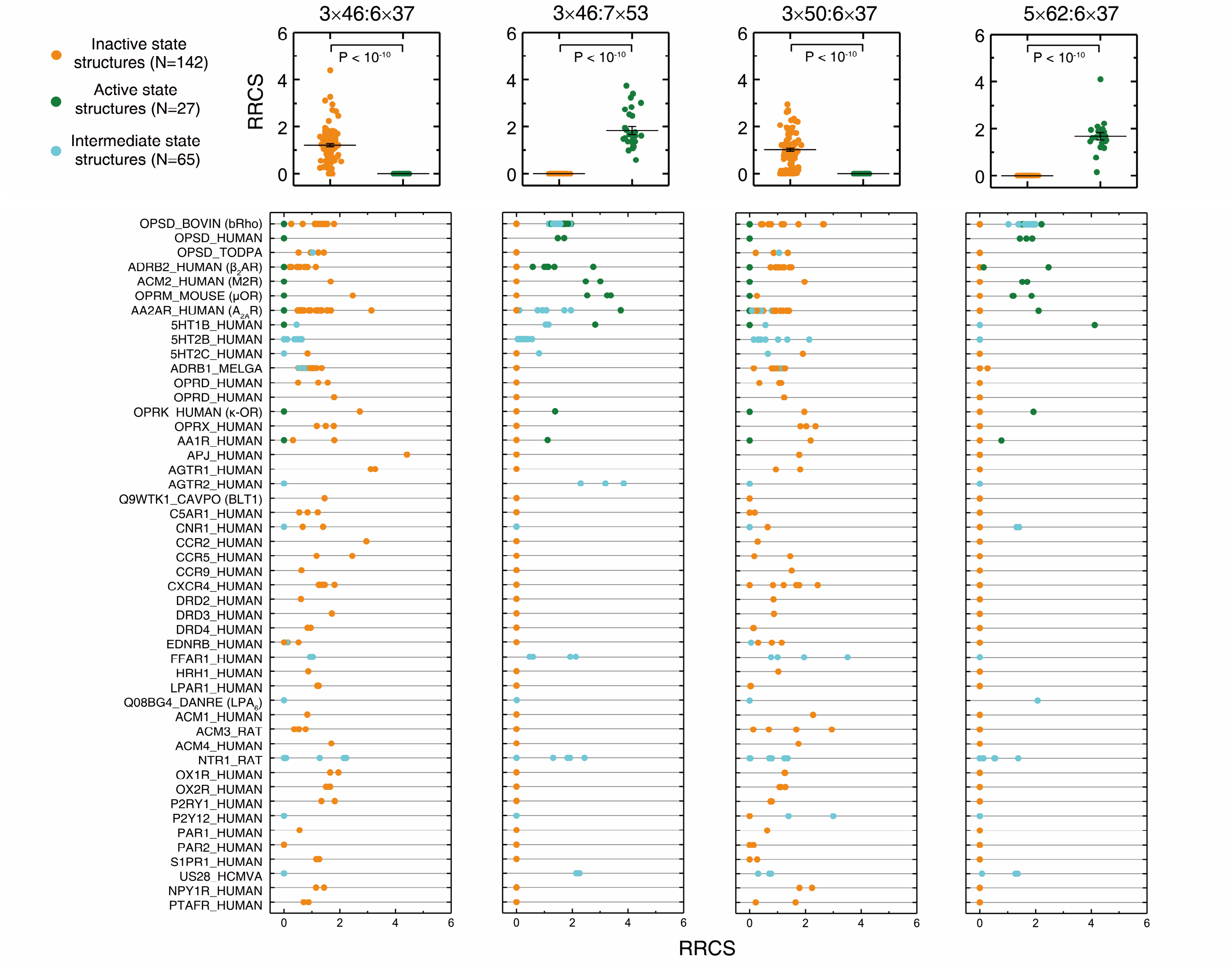

Fig. S5. Microswitch residues (3× 46, 6 ×37) share conserved rearrangements of residue contacts upon activation. These residue pairs have significant different RRCS between inactive- and active- state, i.e., they evolve similar rearrangements of residue contacts upon activation (two-sample *t*-test, error bars represent the standard error of the mean). The comparison of RRCS between inactive- and active- state were plotted in receptor-specific manner for 45 class A GPCRs with determined structures, where inactive, active and intermediate states are coloured in orange, cyan and green, respectively.

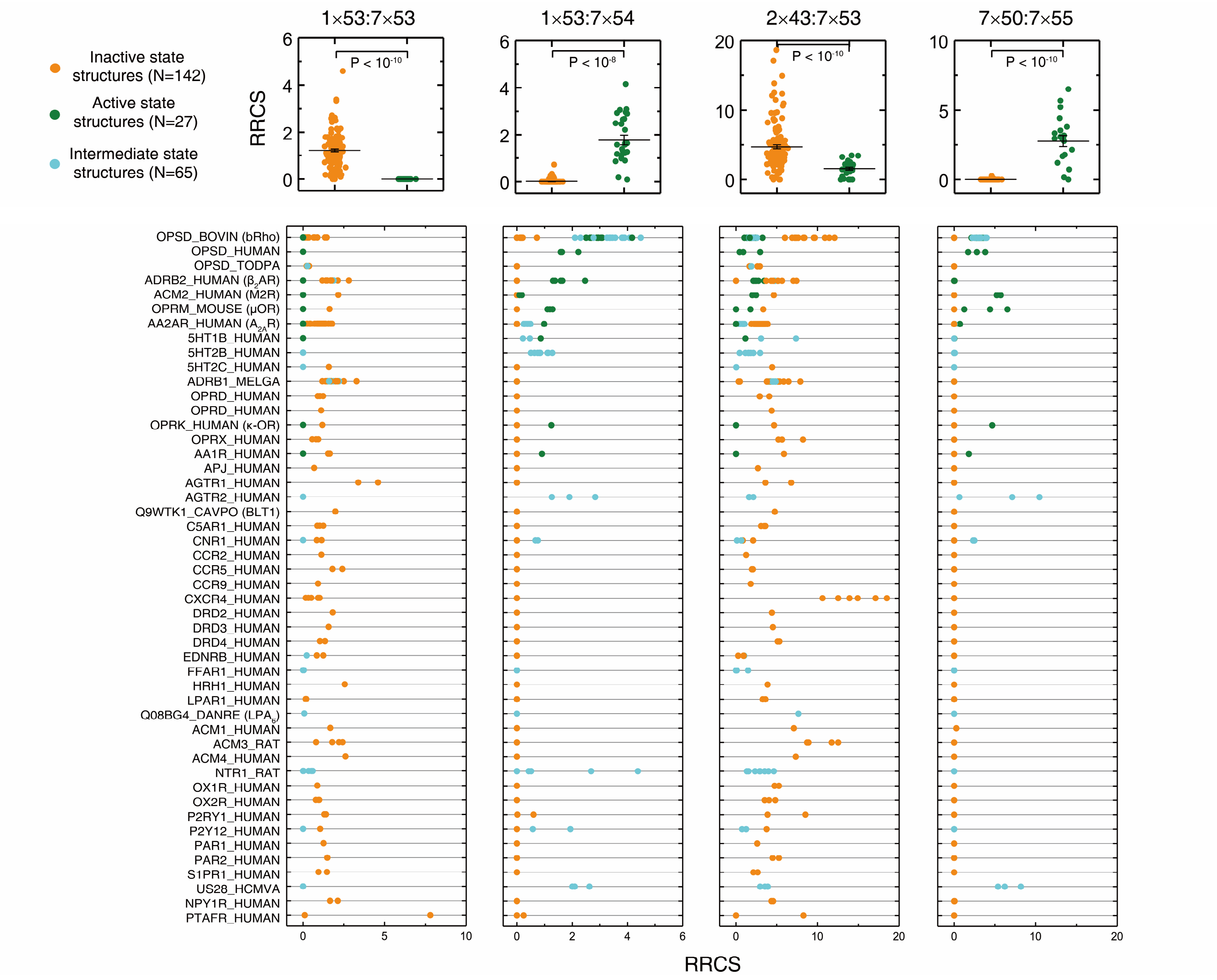

Fig. S6. Translocation of $Y^{7\times53}$ during receptor activation. These residue pairs around $Y^{7\times53}$ have significant different RRCS between inactive- and active- states, i.e., they evolve similar rearrangements of residue contacts upon activation (two-sample *t*-test, error bars represent the standard error of the mean). The comparison of RRCS between inactive- and active- state were plotted in receptor-specific manner for 45 class A GPCRs with determined structures, where inactive, active and intermediate states are coloured in orange, cyan and green, respectively.

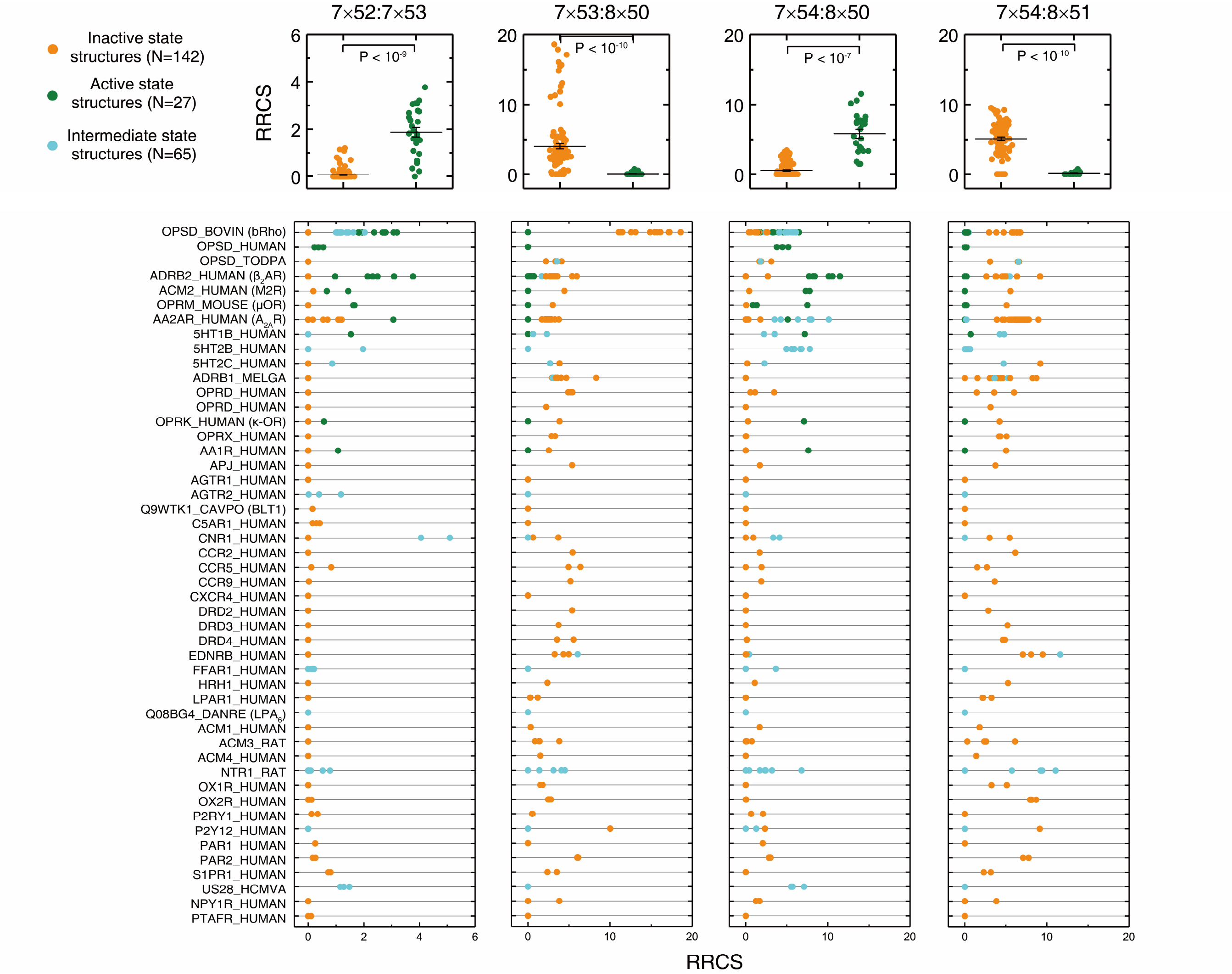

Fig. S7. Residue pairs around Y$^{7\times53}$ share conserved rearrangements of residue contacts upon activation. These residue pairs have significant different RRCS between inactive- and active- state, i.e., they evolve similar rearrangements of residue contacts upon activation (two-sample *t*-test, error bars represent the standard error of the mean). The comparison of RRCS between inactive- and active- state were plotted in receptor-specific manner for 45 class A GPCRs with determined structures, where inactive, active and intermediate states are coloured in orange, cyan and green, respectively.

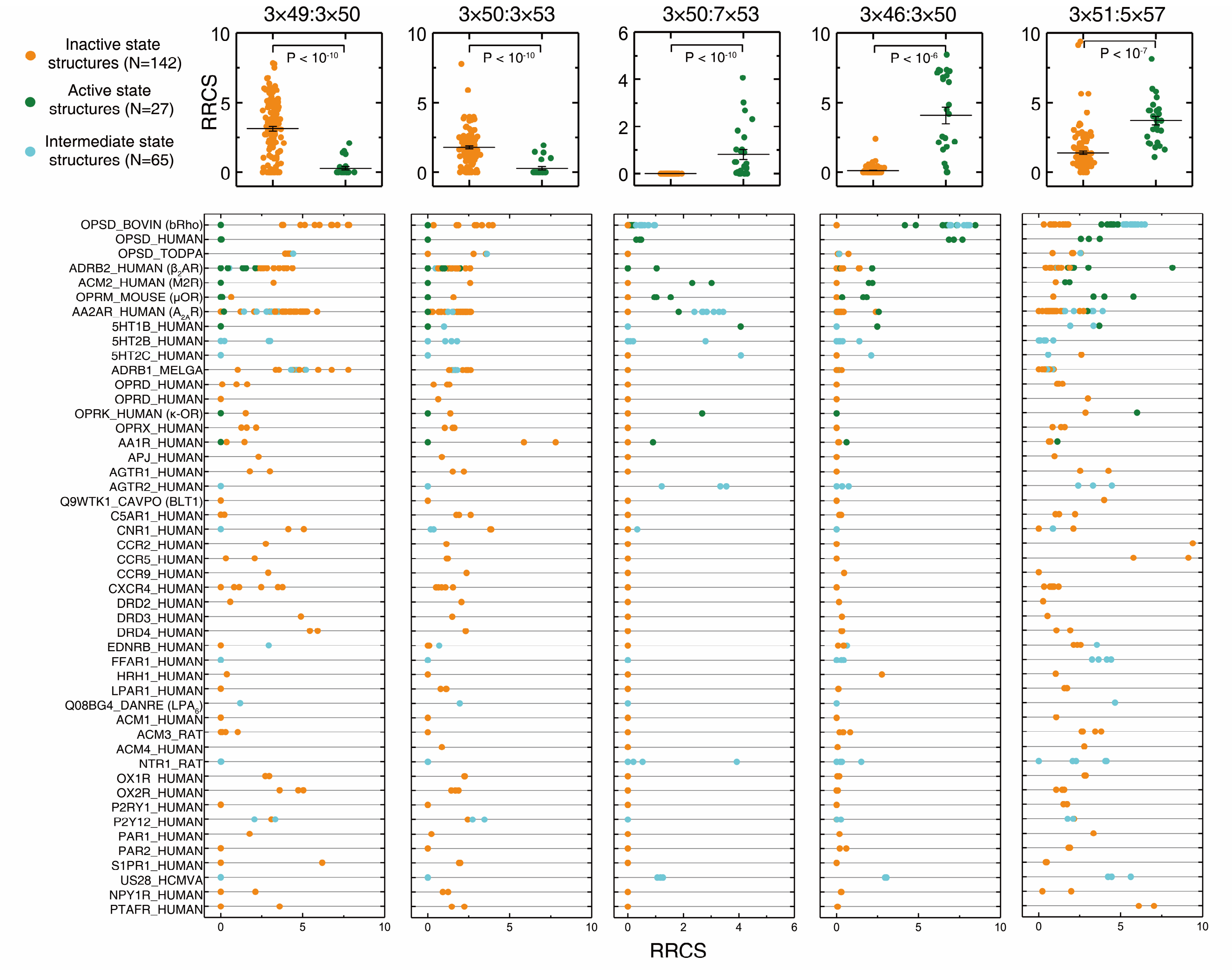

Fig. S8. Residue pairs around G protein-coupling region share conserved rearrangements of residue contacts upon activation. These residue pairs have significant different RRCS between inactive- and active- state, i.e., they evolve rearrangements of residue contacts upon activation (two-sample *t*-test, error bars represent the standard error of the mean). The comparison of RRCS between inactive- and active- state were plotted in receptor-specific manner for 45 class A GPCRs with determined structures, where inactive, active and intermediate states are coloured in orange, cyan and green, respectively.
