# Supplementary material for: Common activation mechanism of class A GPCRs
Source: eLife. 2019 Dec 19;8:e50279. doi: 10.7554/eLife.50279 (PMC6954041; doi:10.7554/eLife.50279)
Supplement: Figure 6—source data 1. [file elife-50279-fig6-data1.docx]

**Figure 6—source data 2. Analysis of the 14 unsuccessful predictions of A_2A_R CAMs/CIMs.** ΔStability (>0 means destabilized; <0 means stabilized) is the change of receptor stability when a mutation was introduced, calculated by Residue Scanning module in BioLuminate^193^. WT, wild-type.

| Position | Mutation | Effect on inactive state (3EML) | Effect on active state (5G53) | Prediction | Experiment result | Discussion |
| --- | --- | --- | --- | --- | --- | --- |
| **Unsuccessful prediction of 9 CAMs** | | | | | | |
| 3×46 | I98N | ΔStability >0 | ΔStability >0, H-bonds with F44^2×42^ | Stabilizes active state | Low expression | May affect receptor folding or trafficking. |
| 3×46 | I98E | ΔStability >0 | ΔStability >0, salt bridge with R102^3×50^ | Stabilizes active state | CIM, >20-fold decrease in EC_50_ | May affect G protein coupling interface |
| 3×49 | D101S | ΔStability >0 | ΔStability <0, H-bonds with Y112^34×53^ | Breaks the restrains with R102^3×50^ | Close to WT | May affect G protein coupling interface |
| 3×50 | R102H | ΔStability >0 | ΔStability >0, salt bridge with D101^×49^ | Stabilizes active state | Close to WT | May affect G protein coupling interface |
| 3×51 | Y103E | ΔStability >0, salt bridge w/R107^3×55^ | ΔStability >0, salt bridge with R199^5×60^ | Stabilizes TM5-TM6 contacts | Close to WT | May have indirect impact or no effect on TM6 rotation |
| 6×40 | I238Q | ΔStability >0 | ΔStability >0, H-bonds with R102^3×50^ and R291^7×56^ | Stabilizes active state | Close to WT | Increases TM3-TM6 contacts, but may not affect the rotation of TM6 |
| 6×40 | I238E | ΔStability >0 | ΔStability >0, salt bridge with R102^3×50^ | Stabilizes active state | Close to WT | Increases TM3-TM6 contacts, but may not affect the rotation of TM6 |
| 6×40 | I238A | ΔStability >0, less hydrophobic contacts | ΔStability >0, less hydrophobic contacts | Loosens TM3-TM6 contacts | Close to WT | Destabilizes both inactive and active states, but may not affect the rotation of TM6 |
| 7×45 | N280S | ΔStability >0 | ΔStability >0, H-bonds with W246^6×48^ | Stabilizes active state | Close to WT | Destabilizes both inactive and active states, but may not affect the rotation of TM6 |
| **Unsuccessful prediction of 5 CIMs** | | | | | | |
| 3×40 | I92Y | ΔStability >0, stacking w/F242^6×44^ | ΔStability >0, side chains rotate away from F242^6×44^ | Tightens TM3-6 contacts | Close to WT, slightly high basal activity | Makes the rotation of the cytoplasmic end of TM6 easier in active state |
| 3×50 | R102A | ΔStability >0 | ΔStability >0, affect G protein coupling interface | Reduces interaction with G protein | Close to WT | A102^3×50^ doesn’t affect G protein coupling for A_2A_R |
| 6×40 | I238M | ΔStability >0, more hydrophobic contacts | ΔStability <0, more hydrophobic contacts | Tightens TM3-6 contacts | Close to WT | May stabilize the active state, but may not affect the rotation of TM6 |
| 6×44 | F242T | ΔStability >0 | ΔStability >0, may greatly affect signal initiation | May block the rotation of TM6 | Close to WT | T242^6×44^ doesn’t affect signal initiation for A_2A_R |
| 6×44 | F242L | ΔStability >0 | ΔStability >0, may greatly affect signal initiation | May block the rotation of TM6 | Close to WT | L242^6×44^ doesn’t affect signal initiation for A_2A_R |
