# Supplementary material for: Common activation mechanism of class A GPCRs
Source: eLife. 2019 Dec 19;8:e50279. doi: 10.7554/eLife.50279 (PMC6954041; doi:10.7554/eLife.50279)
Supplement: Figure 6—source data 2. — ΔStability (>0 means destabilized;<0 means stabilized) is the change of receptor stability when a mutation was introduced, calculated by Residue Scanning module in BioLuminate (Beard et al., 2013). WT, wild-type. [file elife-50279-fig6-data2.docx]

**Figure 6—source data 1.** **Functional and ligand binding properties of A_2A_R mutations.**

|  | Layer | Position | Mutation | Expression  (% WT) | Binding assay  IC_50_ (nM) | Function assay (cAMP accumulation) | | |
| --- | --- | --- | --- | --- | --- | --- | --- | --- |
|  |  |  |  |  |  | Basal activity  (% WT) | EC_50_ (nM) | Fold change in  agonist potency |
| WT | | | | 100 | 318.7 ± 41.2 | 100 | 78.4 ± 22.5 |  |
| CAM | 1 | 6×44 | F242A | 92.0 ± 10.3 | 120.8 ± 17.8 | 196.5 ± 38.9 | 10.4 ± 1.4 | 7.5-fold increase |
|  |  | 3×40 | I92N | 66.7 ± 6.4 | 43.8 ± 10.5 | 735.1 ± 131.8 | N.D.* | Constitutively active |
|  | 2 | 3×43 | L95A | 96.7 ± 14.2 | 91.07 ± 53.0 | 2845.0 ± 738.6 | N.D.* |  |
|  |  | 3×43 | L95R | 42.3 ± 4.4 | 230.6 ± 57.1 | 224.8 ± 44.4 | N.D.* |  |
|  |  | 6×40 | I238Y | 43.0 ± 2.1 | 159.8 ± 33.6 | 1074.9 ± 81.1 | N.D.* |  |
|  | 4 | 3×49 | D101N | 41.7 ± 4.6 | 147.5 ± 99.4 | 840.6 ± 280.1 | N.D.* |  |
| CIM | 1 | 2×50 | D52A | 46.3 ± 1.7 | 154.6 ± 49.4 | 79.7 ± 7.8 | N.D.^†^ | Completely abolished |
|  |  | 3×40 | I92A | 90.0 ± 8.6 | 440.3 ± 240.3 | 70.7 ± 2.2 | 593.6 ± 66.4 | 7.6-fold decrease |
|  |  | 6×44 | F242R | 95.0 ± 2.7 | 894.0 ± 214.7 | 63.1 ± 12.6 | 29304.3 ± 12950.3 | 373.6-fold decrease |
|  |  | 6×48 | W246A | 116.0 ± 12.5 | 21672.5 ± 5153.4 | 80.9 ± 9.2 | 17247.5 ± 3625.9 | 219.9-fold decrease |
|  |  | 7×45 | N280R | 87.7 ± 8.7 | 700.2 ± 225.7 | 78.0 ± 9.3 | N.D.^†^ | Completely abolished |
|  | 2 | 2×46 | L48R | 63.7 ± 5.0 | 193.9 ± 50.6 | 93.3 ± 9.1 | N.D.^†^ | Completely abolished |
|  |  | 3×43 | L95F | 98.7 ± 22.6 | 223.1 ± 79.3 | 93.2 ± 20.0 | 609.5 ± 42.1 | 7.8-fold decrease |
|  |  | 6×41 | V239Q | 58.7 ± 4.9 | 60.1 ± 5.7 | 80.4 ± 12.8 | 515.7 ± 30.7 | 6.6-fold decrease |
|  |  | 7×49 | N284A | 116.3 ± 17.0 | 459.1 ± 136.1 | 73.6 ± 11.1 | N.D.^†^ | Completely abolished |
|  |  | 7×49 | N284K | 124.0 ± 24.6 | 5007.3 ± 1279.1 | 69.0 ± 4.3 | N.D.^†^ | Completely abolished |
|  | 3 | 3×46 | I98A | 105.3 ± 15.1 | 818.2 ± 311.8 | 71.8 ± 4.3 | 1821.7 ± 513.3 | 23.2-fold decrease |
|  |  | 6×37 | L235A | 100.7 ± 11.1 | 211.3 ± 181.8 | 71.1 ± 8.5 | 298.6 ± 95.8 | 3.8-fold decrease |
|  |  | 7×53 | Y288A | 63.3 ± 6.2 | 159.7 ± 50.2 | 102.6 ± 12.8 | 1262.2 ± 188.2 | 16.1-fold decrease |
|  |  | 7×54 | A289F | 122.3 ± 13.9 | 75.6 ± 3.6 | 76.0 ± 10.0 | 4706.7 ± 644.1 | 60.0-fold decrease |
|  | 4 | 3×50 | R102L | 90.3 ± 4.8 | 33.9 ± 6.3 | 79.95 ± 29.6 | 789.1 ± 63.2 | 10.1-fold decrease |

*Basal activity was too high to determine an accurate EC_50_ value.

^†^No stimulation of cAMP production was observed with 50 μM CGS21680.
