# Supplementary material for: Common activation mechanism of class A GPCRs
Source: eLife. 2019 Dec 19;8:e50279. doi: 10.7554/eLife.50279 (PMC6954041; doi:10.7554/eLife.50279)
Supplement: Supplementary file 1. [file elife-50279-supp1.docx]

**Supplementary File**: Key Resources Table

| **Reagent type (species) or resource** | **Designation** | **Source or reference** | **Identifiers** | **Additional information** |
| --- | --- | --- | --- | --- |
| gene (Drosophila melanogaster) | ADORA2A |  | HGNC:HGNC:263 |  |
| gene (Drosophila melanogaster) | HTR1B |  | HGNC:HGNC:5287 |  |
| strain, strain background (Escherichia coli) | DH5α | TIANGEN | CB101 | chemically competent cells |
| genetic reagent (Homo-sapiens) | Site-directed mutagenesis kit | SBS Genetech Co,  Ltd | SDM-15 |  |
| cell line (Homo-sapiens) | HEK-293 | ATCC | CRL-1573 | Cell lines were tested periodically for mycoplasma contamination |
| cell line (Homo-sapiens) | HEK-293T | obtained from and authenticated by the Cell Bank at the Chinese Academy of Science |  | Cell lines were tested periodically for mycoplasma contamination |
| transfected construct (human) | Lipofectamine 2000 transfection reagent | Invitrogen |  |  |
| antibody | anti-Flag, M2 (Mouse monoclonal) | Sigma-Aldrich | Cat#  F3165, | FACS (1:300) |
| antibody | anti-HA, (Mouse monoclonal) | Cell Signaling Technology | Cat#  2367 | FACS (1:1000) |
| antibody | anti-mouse Alexa-488-conjugated secondary antibody | Cell Signaling Technology, Danvers, MA, USA | Cat#  4408 | FACS (1:1000) |
| recombinant DNA reagent | hA_2A_AR-plasmid DNA (FLAG-tag at N–terminus, in pcDNA3.1) | Massink et al. 2015 PMID: 25473121 |  |  |
| recombinant DNA reagent | HTR1B-HA | The cDNA Resource Center | Cat#HTR01BTN00 |  |
| recombinant DNA reagent | HTR7 | Addgene | #1000000068 |  |
| recombinant DNA reagent | pcDNA3.1 vector | Invitrogen, Carlsbad, CA, USA | V79520 |  |
| recombinant DNA reagent | pRluc8-N1 vector | Lan et al. 2011  PMID: 22816793 |  |  |
| recombinant DNA reagent | Nluc-EPAC-VV | Masuho et al. 2015  PMID: 26628681 |  |  |
| commercial assay or kit | BCA assay kit | Pierce Biotechnology, Pittsburgh, PA, USA |  |  |
| commercial assay or kit | PRESTO-Tango Kit | Addgene | Cat#  1000000068 |  |
| commercial assay or kit | Muta-directTM kit | Beijing SBS Genetech Co., Ltd., China | SDM-15 |  |
| commercial assay or kit | Quikchange mutagenesis kit | Agilent Technologies, Santa Clara, CA, USA | 200523 |  |
| commercial assay or kit | LANCE Ultra cAMP kit | PerkinElmer | TRF0264 |  |
| chemical compound, drug | [3H]-CGS21680 | PerkinElmer | NET1021250UC |  |
| chemical compound, drug | Dihydroergotamine Mesylate | TCI (Shanghai) Development company | D2633 |  |
| chemical compound, drug | IBMX | Abcone | I72182 |  |
| chemical compound, drug | Forskolin | Abcone | F38265 |  |
| chemical compound, drug | Furimazine NanoGlo substrate | Promega | N1120 |  |
| chemical compound, drug | CGS21680 | Sigma-Aldrich | 119137 |  |
| chemical compound, drug | coelenterazine-h | Nanolight | Cat. #301 |  |
| software, algorithm | Schrödinger Suite 2017-4 | Schrödinger | www.schrodinger.com |  |
| software, algorithm | Prism v.7.0 | GraphPad Software Inc. | N/A |  |
| sequence-based reagent | HTR1B-I137N-F | This paper | PCR primers | GTTGCACTGCCTCCAACCTGCACCTCTGTGTC |
| sequence-based reagent | HTR1B-I137N-R | This paper | PCR primers | GACACAGAGGTG CAGGTTGGAGGC AGTGCAAC |
| sequence-based reagent | HTR1B-I137A-F | This paper | PCR primers | GTTGCACTGCCTCCGCCCTGCACCTCTGTGTC |
| sequence-based reagent | HTR1B-I137A-R | This paper | PCR primers | GACACAGAGGTG CAGGGCGGAGGC AGTGCAAC |
| sequence-based reagent | HTR1B-F323A-F | This paper | PCR primers | ATCATTTTGGGAGCCGCTATTGTGTGTTGGCTACC |
| sequence-based reagent | HTR1B-F323A-R | This paper | PCR primers | GGTAGCCAA CACACAATA GCGGCTCCC AAAATGAT |
| sequence-based reagent | HTR1B-F323H-F | This paper | PCR primers | ATCATTTTGGGAGCCCATATTGTGTGTTGGCTACC |
| sequence-based reagent | HTR1B-F323H-R | This paper | PCR primers | GGTAGCCAA CACACAATA TGGGCTCCC AAAATGAT |
| sequence-based reagent | A2AR-F242A-F | This paper | PCR primers | TCATTGTGG GGCTCGCTG CCCTCTGCT GGCTGCCC |
| sequence-based reagent | A2AR-F242A-R | This paper | PCR primers | GGGCAGCCA GCAGAGGGC AGCGAGCCC CACAATGA |
| sequence-based reagent | A2AR-I92N-F | This paper | PCR primers | TCACGCAGA GCTCCAACT TCAGTCTCC TGGCCA |
| sequence-based reagent | A2AR-I92N-R | This paper | PCR primers | TGGCCAGGA GACTGAAGT TGGAGCTCT GCGTGA |
| sequence-based reagent | A2AR-L95A-F | This paper | PCR primers | GCTCCATCT TCAGTGCCC TGGCCATCG CCA |
| sequence-based reagent | A2AR-L95A-R | This paper | PCR primers | TGGCGATGG CCAGGGCAC TGAAGATGG AGC |
| sequence-based reagent | A2AR-L95R-F | This paper | PCR primers | CTCCATCTTCAGTCGC  CTGGCCATCGCCA |
| sequence-based reagent | A2AR-L95R-R | This paper | PCR primers | TGGCGATGGCCAGGCG  ACTGAAGATGGAG |
| sequence-based reagent | A2AR-I238Y-F | This paper | PCR primers | GTCAGCGGC CATCTATGT GGGGCTCTT TGCCCTC |
| sequence-based reagent | A2AR-I238Y-R | This paper | PCR primers | GAGGGCAAA GAGCCCCAC ATAGATGGC CGCTGA C |
| sequence-based reagent | A2AR-D101N-F | This paper | PCR primers | GGCCATCGC CATTAACCG CTACATTGC CATCCG |
| sequence-based reagent | A2AR-D101N-R | This paper | PCR primers | CGGATGGCA ATGTAGCGG TTAATGGCG ATGGCC |
| sequence-based reagent | A2AR-D52A-F | This paper | PCR primers | GTCACTGGC GGCGGCCGC CATCGCAGT GGGTGTG |
| sequence-based reagent | A2AR-D52A-R | This paper | PCR primers | CACACCCAC TGCGATGGC GGCCGCCGC CAGTGAC |
| sequence-based reagent | A2AR-I92A-F | This paper | PCR primers | TCACGCAGA GCTCCGCCT TCAGTCTCC TGGCCA |
| sequence-based reagent | A2AR-I92A-R | This paper | PCR primers | TGGCCAGGA GACTGAAGG CGGAGCTCT GCGTGA |
| sequence-based reagent | A2AR-F242R-F | This paper | PCR primers | TCATTGTGG GGCTCCGTG CCCTCTGCT GGCTGCCC |
| sequence-based reagent | A2AR-F242R-R | This paper | PCR primers | GGGCAGCCA GCAGAGGGC ACGGAGCCC CACAATGA |
| sequence-based reagent | A2AR-W246A-F | This paper | PCR primers | TCTTTGCCC TCTGCGCGC TGCCCCTAC ACAT |
| sequence-based reagent | A2AR-W246A-R | This paper | PCR primers | ATGTGTAGG GGCAGCGCG CAGAGGGCA AAGA |
| sequence-based reagent | A2AR-N280R-F | This paper | PCR primers | CATCGTCCTCTCCCACACCAGG  TCGGTTGTGAATCCC |
| sequence-based reagent | A2AR-N280R-R | This paper | PCR primers | GGGATTCACAACCGACCTGGTG  TGGGAGAGGACGATG |
| sequence-based reagent | A2AR-L48R-F | This paper | PCR primers | TGTGGTGTCACGGGCGGCGGCCG |
| sequence-based reagent | A2AR-L48R-R | This paper | PCR primers | CGGCCGCCGCCCGTGACACCACA |
| sequence-based reagent | A2AR-L95F-F | This paper | PCR primers | GCTCCATCT TCAGTTTCC TGGCCATCG CCA |
| sequence-based reagent | A2AR-L95F-R | This paper | PCR primers | TGGCGATGG CCAGGAAAC TGAAGATGG AGC |
| sequence-based reagent | A2AR-V239Q-F | This paper | PCR primers | GCCAAGTCACTGGCCATCATT  CAGGGGCTCTTTG |
| sequence-based reagent | A2AR-V239Q-R | This paper | PCR primers | CAAAGAGCCCCTGAATGA  TGGCCAGTGACTTGGC |
| sequence-based reagent | A2AR-N284A-F | This paper | PCR primers | GTAGGCGTAGATGAAGGGAGC  CACAACCGAATTGGTGTGG |
| sequence-based reagent | A2AR-N284A-R | This paper | PCR primers | CCACACCAATTCGGTTGTGGC  TCCCTTCATCTACGCCTAC |
| sequence-based reagent | A2AR-N284K-F | This paper | PCR primers | GGCGTAGATGAAGGGCTTCA  CAACCGAATTGGTGT |
| sequence-based reagent | A2AR-N284K-R | This paper | PCR primers | ACACCAATTCGGTTGTGAAG  CCCTTCATCTACGCC |
| sequence-based reagent | A2AR-I98A-F | This paper | PCR primers | CAGTCTCCT GGCCGCCGC CATTGACCG CTA C |
| sequence-based reagent | A2AR-I98A-R | This paper | PCR primers | GTAGCGGTC AATGGCGGC GGCCAGGAG ACTG |
| sequence-based reagent | A2AR-L235A-F | This paper | PCR primers | GCTGCCAAG TCAGCGGCC ATCATTGTG GGG C |
| sequence-based reagent | A2AR-L235A-R | This paper | PCR primers | GCCCCACAA TGATGGCCG CTGACTTGG CAGC |
| sequence-based reagent | A2AR-Y288A-F | This paper | PCR primers | TTGTGAATCCCTTCATCG  CCGCCTACCGTATCCGCG |
| sequence-based reagent | A2AR-Y288A-R | This paper | PCR primers | CGCGGATACGGTAGGCGG  CGATGAAGGGATTCACAA |
| sequence-based reagent | A2AR-A289F-F | This paper | PCR primers | TGTGAATCCCTTCATCTACT  TCTACCGTATCCGCGAGTTC |
| sequence-based reagent | A2AR-A289F-R | This paper | PCR primers | GAACTCGCGGATACGGTAGA  AGTAGATGAAGGGATTCACA |
| sequence-based reagent | A2AR-R102L-F | This paper | PCR primers | GGCCATCGC CATTGACCT CTACATTGC CATCCGC |
| sequence-based reagent | A2AR-R102L-R | This paper | PCR primers | GCGGATGGC AATGTAGAG GTCAATGGC GATGGCC |
| sequence-based reagent | 5HT7-L173A-F/R | This paper | gBlock ds primer | CCTCGTAGTAATCTCAGTTTGTTTTGTAAAAAAACTCCGGCAGCCCAGTAACTACCTGATCGTCTCACTCGCACTGGCCGATCTCAGTGTGGCAGTCGCTGTTATGCCGTTCGTCTCTGTCACAGACCTGATAGGTGGTAAGTGGATCTTCGGACATTTTTTTTGTAACGTGTTTATTGCGATGGACGTTATGTGTTGCACCGCCTCAATTATGACTGCCTGTGTGATCAGTATCGACAGATACTTGGGCATTACACGCCCCTTGACATACCCAGTGAGACAAAATGGAAAGTGCATGGCCAAGATGATTCTC |
| sequence-based reagent | 5HT7-F336R-F/R | This paper | gBlock ds primer | GTTCATGTACTATCAGATCTATAAGGCGGCTCGAAAGAGTGCCGCCAAACATAAGTTTCCGGGGTTCCCCAGAGTGGAGCCCGATAGCGTAATCGCACTGAACGGTATTGTGAAACTGCAGAAAGAAGTGGAAGAGTGTGCGAATCTCTCCAGACTGCTTAAGCATGAGCGAAAAAATATCTCCATCTTCAAGAGGGAACAAAAAGCCGCCACCACCCTCGGTATCATCGTGGGAGCGCGCACGGTGTGTTGGCTGCCTTTCTTCCTCCTGTCCACTGCTAGACCATTCATTTGCGGAACTTCCTGCTCTTGTATTCCGCTGTGGGTTGAAAGGACCTTTCTGTGGCTGGGTTACGCTAACTCACTGATTAATCCATTCATTTACGCATTTTTCAACCGGGACCTGAGGACCA |
| sequence-based reagent | 5HT7-N380K-F/R | This paper | gBlock ds primer | GTTCATGTACTATCAGATCTATAAGGCGGCTCGAAAGAGTGCCGCCAAACATAAGTTTCCGGGGTTCCCCAGAGTGGAGCCCGATAGCGTAATCGCACTGAACGGTATTGTGAAACTGCAGAAAGAAGTGGAAGAGTGTGCGAATCTCTCCAGACTGCTTAAGCATGAGCGAAAAAATATCTCCATCTTCAAGAGGGAACAAAAAGCCGCCACCACCCTCGGTATCATCGTGGGAGCGTTCACGGTGTGTTGGCTGCCTTTCTTCCTCCTGTCCACTGCTAGACCATTCATTTGCGGAACTTCCTGCTCTTGTATTCCGCTGTGGGTTGAAAGGACCTTTCTGTGGCTGGGTTACGCTAACTCACTGATTAAACCATTCATTTACGCATTTTTCAACCGGGACCTGAGGACCACCTACAGATCCTTGTTGCAGTGCCAGTACCGGAACATAAACCGCAAACTTTCAGCCGCCGGAATGCACGAAGCCCTGAAACTGGCCG |
